# PeSV-Fisher: Identification of Somatic and Non-Somatic Structural Variants Using Next Generation Sequencing Data

**DOI:** 10.1371/journal.pone.0063377

**Published:** 2013-05-21

**Authors:** Geòrgia Escaramís, Cristian Tornador, Laia Bassaganyas, Raquel Rabionet, Jose M. C. Tubio, Alexander Martínez-Fundichely, Mario Cáceres, Marta Gut, Stephan Ossowski, Xavier Estivill

**Affiliations:** 1 Genetic Causes of Disease Group, Centre for Genomic Regulation (CRG), Barcelona, Spain; 2 Universitat Pompeu Fabra (UPF), Barcelona, Spain; 3 Centro de Investigación Biomédica en Red en Epidemiología y Salud Pública (CIBERESP), Barcelona, Spain; 4 Hospital del Mar Medical Research Institute (IMIM), Barcelona, Spain; 5 Galician Foundation of Genomic Medicine-SERGAS, Complexo Hospitalitario Universitario de Santiago (CHUS), Santiago de Compostela, Spain; 6 Institut de Biotecnologia i de Biomedicina, Universitat Autònoma de Barcelona, Bellaterra, Spain; 7 Institució Catalana de Recerca i Estudis Avançats (ICREA), Barcelona, Spain; 8 National Center of Genomic Analysis (CNAG-CRG), Barcelona, Spain; 9 Genomic and Epigenomic Variation in Disease Group, Centre for Genomic Regulation (CRG), Barcelona, Spain; Seoul National University College of Medicine, Republic of Korea

## Abstract

Next-generation sequencing technologies expedited research to develop efficient computational tools for the identification of structural variants (SVs) and their use to study human diseases. As deeper data is obtained, the existence of higher complexity SVs in some genomes becomes more evident, but the detection and definition of most of these complex rearrangements is still in its infancy. The full characterization of SVs is a key aspect for discovering their biological implications. Here we present a pipeline (*PeSV-Fisher*) for the detection of deletions, gains, intra- and inter-chromosomal translocations, and inversions, at very reasonable computational costs. We further provide comprehensive information on co-localization of SVs in the genome, a crucial aspect for studying their biological consequences. The algorithm uses a combination of methods based on paired-reads and read-depth strategies. *PeSV-Fisher* has been designed with the aim to facilitate identification of somatic variation, and, as such, it is capable of analysing two or more samples simultaneously, producing a list of non-shared variants between samples. We tested *PeSV-Fisher* on available sequencing data, and compared its behaviour to that of frequently deployed tools (BreakDancer and VariationHunter). We have also tested this algorithm on our own sequencing data, obtained from a tumour and a normal blood sample of a patient with chronic lymphocytic leukaemia, on which we have also validated the results by targeted re-sequencing of different kinds of predictions. This allowed us to determine confidence parameters that influence the reliability of breakpoint predictions.

**Availability:**

*PeSV-Fisher* is available at http://gd.crg.eu/tools.

## Introduction

Genomic structural variations (SVs) include copy number variants (CNVs) of genomic segments (typically >100 bp), insertions, and balanced rearrangements, such as inversions and translocations [1], [2]. The fraction of the genome affected by SVs is comparatively larger than that accounted for by single nucleotide polymorphisms (SNPs) and other smaller scale variants (Indels) [3]; currently, around 15% of the human genome is considered to fall into copy number variable regions [4]. Thus, their potential role in the contribution to genetic differences between individuals and species, to genetic diseases, and to the somatic differences between normal and cancer cells has become increasingly apparent [1]–[3], [5], [6].

Advances in DNA sequencing technologies have enabled the exploration of genomic SVs at a very fine scale, allowing a genome-wide characterization of breakpoints for most classes and sizes of SVs at base-pair resolution. However, the nature and the huge amount of next-generation sequencing (NGS) data pose substantial computational and bioinformatics challenges. Four main strategies exist for the detection of SVs breakpoints from NGS data (reviewed in [7], [8]). Briefly, these strategies are: (1) Paired-reads (PR) approaches, based on the alignment of sequence pairs corresponding to both ends of a clone or a DNA fragment, which find clusters of aberrantly mapped pairs of reads that suggest the presence of SVs [9]–[12]; (2) read-depth (RD) analysis, that detect CNVs by analysing the density of reads mapped to a given interval of the reference genome [13]–[15]; (3) split-read (SR) [16] and clip-read (CR) [17] analysis, which directly identify sequence reads that contain breakpoints of SVs; and (4) sequence assembly (AS), which enable the fine-scale discovery of SVs, including novel (non-reference) sequence insertions [5], [18], [19].

Each of the above-mentioned approaches has limits in terms of the type and size of SVs that they are able to detect, as each one has different strengths and weaknesses [7], [8]. For example, PR approaches have difficulties in mapping assignments when the read pairs fall into repetitive regions or when there are SNPs or other sequence features mapping close to the breakpoints. Furthermore, the size of insertions that can be detected by PR is limited by the library’s insert size mean and standard deviation. Although RD analysis is the only sequencing-based method to accurately predict absolute copy-numbers [13], [20], its breakpoint resolution is poor. RD is further hampered by PCR induced coverage biases and is unable to detect copy-number neutral variants such as inversions and balanced translocations. The SR strategy has had a limited application in the analysis of NGS data due to the difficulty of aligning short reads (reliable only in unique genomic regions), algorithmic issues with aligning across large gaps and the need of a higher depth of coverage in order to obtain sufficient split reads matching the breakpoints. Finally, AS approaches require higher computational costs, are very-time consuming, and are still prone to assembly errors.

So far, there is no single SV caller with the capacity to detect the full range of changes, since most of the existing methods for SV prediction use only one type of the described approaches. Although the PR strategy would, *a priori,* be able to detect most types of SV, available methods designed to detect SV by PR focus mainly on the discovery of deletions, insertions and inversions. As an alternative, algorithms like SVMerge [21] propose to integrate multiple existing SVs algorithms, which could complement each other and enhance their capabilities for SVs detection. However, the computational requirements for such a process are very high.

More recent methods have begun to consider both PR and RD signals [22]–[24], although most of them have focused only on detection of CNVs, with the exception of the recently described GASVPro algorithm that combines these two strategies for the detection of CNVs and inversions [24]. Only the HYDRA [25] and the next-generation VariationHunter [26] algorithms have moved one step further, and offer the capability to predict retrotransposition events. Additionally, two other methods, Pindel and Delly, have considered the combination of PR and SR signals. Pindel [16] uses both strategies for the detection of large deletions and medium-sized insertions, while Delly [27] integrates PR and SR for the characterization of balanced and unbalanced structural variants.

In the last years, the systematic whole-genome sequencing of several cancer samples and germline genomes has revealed a high level of structural genomic complexity in some populations of tumour or germline cells [28]–[31]. This high genomic complexity consists of a specific pattern of breakpoint accumulation for different types of SVs (deletions, duplications, intra- and inter-chromosomal translocations, and inversions) occurring in relatively small focal regions, involving one or several chromosomes [28]. At the genomic level, this phenomenon demonstrates the importance of the capability to predict the complete spectrum of SV types and to correctly interpret these predictions.

Here we present a novel computational tool to detect and interpret five general types of SVs (deletions, copy-number gains, intra- and inter-chromosomal translocations, and inversions) present in a given genome using NGS data. NGS analysis generates a large amount of SV predictions, and therefore it is essential for computational tools to try to obtain the most reliable set of potential variants in order to facilitate the downstream biological validation. In this sense, our pipeline, called *PeSV-Fisher*, is based on a combinatorial analysis of PR and RD strategies that lead to the detection and correct interpretation of simple and complex structural rearrangements. In addition, for all those breakpoints predicted by PR that are not supported by RD or defined as complex SV, our pipeline includes a module that filters out rearrangement predictions involving multi-copy elements such as segmental duplications (SDs), simple sequence repeats (SSRs) or transposable elements (TEs).

*PeSV-Fisher* has been designed with the aim of facilitating the identification of somatic variation, and, as such, it is capable of analysing two or more samples simultaneously, producing a list of non-shared variants between tumour and normal samples, although it can also analyse samples individually.

## Algorithm Description

*PeSV-Fisher* works on the two different types of sequence data based on pairs of reads, namely paired-end or mate-pair libraries. Paired-end libraries are constructed with small insert sizes (<600 bp) generated by the fragmentation of genomic DNA into short segments followed by gel-based size selection and sequencing of both ends of the segments. In contrast, mate-pair (jumping) libraries are constructed with larger insert sizes (>2 kb). The DNA is fragmented and adaptors are added at the ends, then it is circularized and randomly sheared, generating smaller fragments; the fragment containing the adaptors is selected, and its ends are sequenced [9]. Computationally, the main difference between the two procedures is the strand order of the resulting aligned pair of reads. However, for readability, from now on we will assume that the data has been generated using paired-end libraries. The pipeline starts from sequence alignment data in the BAM format, a quasi standard widely accepted in the sequencing community and produced by most NGS alignment tools. This way, the users can apply their alignment algorithm of choice, such as MAQ [32], SOAP [33], bowtie2 [34] or BWA [35].

The *PeSV-Fisher* toolkit is a compendium of modules performing the following steps: definition of anomalous read-pairs, clustering procedure and breakpoint prediction, read depth analysis, definition and interpretation of structural variants, and filtering structural variant calls (FinalCountDown). These modules are described in detail in the following subsections.

### 1.1 Defining Anomalous Read-pairs

First, read-pairs (RPs) are extracted from each BAM file using SAMtools [36]. Unpaired RPs (i.e. one-end anchored RPs) and orphan RPs (i.e. unmapped RPs) are retained apart.

Second, RPs are assigned to four different categories based on the following criteria: (1) RPs mapped in right order and right orientation (i.e., the leftmost read should be aligned in the forward strand and the rightmost read in the reverse strand); (2) RPs aligned in right orientation but wrong order (i.e., the leftmost read is aligned in the reverse strand and the rightmost in the forward strand); (3) RPs mapped in right order but wrong orientation (i.e., both reads mapped in either forward or reverse strands); and (4) RPs mapped to different chromosomes.

Third, category (1) is used to estimate the empirical distribution of the insert size random variable, *L*. This is used to define the cut-offs to discriminate concordant RPs (those falling into the expected range) from discordant RPs exhibiting a significantly increased or decreased insert size. The empirical distribution of *L* is calculated based on the locations (*left*, *right*) of the innermost RP positions such that *l = right – left,* where *l* correspond to the observations of *L*. Assuming that at least 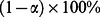 of the RPs are concordant, an upper cut-off, *UC*, and a lower cut-off, *LC*, are calculated as the 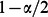 and 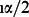 percentiles of the *L* distribution. If *L* is normally distributed, when 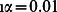, these percentiles correspond to 2.58 standard deviations (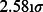) from the mean.

Fourth, the type of aberration is defined based on the characteristics of the alignment. Note that this only classifies the aberration, but it does not attempt to define the type of SV yet. RPs in category (1) with *l* >*UC* are labelled as *distance*-pairs. This type of RPs are usually considered as indicators of deletions, however, as already described by [26] and as we will show later in this section, other types of SVs might be defined by these mapping aberrations. RPs in this category with *l<LC*, which are considered as indicators of insertions, are discarded. The size of the insertions that could be detected from these RPs is limited by the library’s insert size mean and standard deviation, and most PE data does not allow a high-confidence detection of insertions because of this. Detection of novel sequence insertions requires a specific analysis, which is provided by other algorithms [18], [25], [26]. RPs in category (2) will be called *order-*pairs, and are indicators of intra-chromosomal rearrangements. RPs in category (3) will be called *ori-*pairs, and are usually considered as indicators for putative inversions. However, all these RP categories may also be indicators of more complex SVs, which will be described in detail later in this section. Finally, RPs in category (4) will be called *chrpos* or *chrposori-*pairs, depending on whether both reads of the pair are mapped to opposite strands or to the same strand, respectively, and are considered indicators of different inter-chromosomal rearrangements.

### 1.2 Clustering Procedure and Breakpoint Predictions

A clustering procedure is carried out to group indicators pointing to the same aberration. Two different types of clustering approaches are used depending on whether (A) the anomalous RPs consist of paired reads where one read is aligned on the positive DNA strand and the other on the negative strand (such as *distance-*, *order*- or *chrpos*-pairs), or (B) both reads are aligned on the same strand (*ori*- or *chrposori-*pairs).

In case (A) any pair (*i*,*j*) of RPs are clustered together if they meet the following criteria:
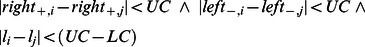
where 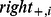 is the rightmost position of the read aligned on the positive strand corresponding to the *i*th RP (likewise for 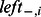). Note that if one assumes that *L* follows a normal distribution, 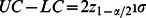, where 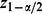 is the 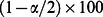 percentile of the cumulative standard normal distribution.

In case (B), the criteria are based on the following conditions:
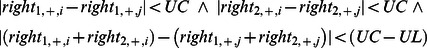
or



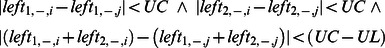
where 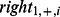 is the rightmost position of read 1 of the *i*th RP, pointing to the 1^st^ breakpoint, and 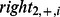 is the rightmost position of read 2, pointing to the 2^nd^ breakpoint, both aligned on the forward strand (likewise for 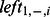 and 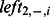 on the reverse strand).

In all cases, any RP that has been included in one cluster is not considered for any other cluster.

Once the clustering procedure is finished, clusters containing at least two RPs are used for breakpoint prediction, and two breakpoints are predicted for each cluster. For reads aligned on the forward strand, putative breakpoints are predicted to fall within the range 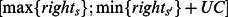, with 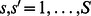, being *S* the number of RPs in a cluster. Likewise, putative breakpoints found with reads aligned on the reverse strand are predicted to fall within the range 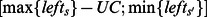.

### 1.3 Somatic Breakpoints

One of the main objectives in cancer genomic studies is the identification of tumour specific variants. In this sense, *PeSV-Fisher* allows the user to analyse paired samples, i.e. tumour and normal cell samples, simultaneously. If the *somatic* option is activated, the algorithm searches for aberrant clusters present in the tumour sample that are not shared with the normal sample, and only these clusters are taken into account in the subsequent analysis modules. Therefore, when a SV is later predicted, it is automatically defined as a somatic SV.

### 1.4 Read Depth Analysis

This module uses both RPs with either one- or two-end anchored reads. The algorithm comprises the following steps executed in a hierarchical order:

First, each chromosome is divided into non-overlapping windows of equal size and the GC content in each window is annotated.

Second, every aligned read is assigned to the window where the highest percentage of its base pairs fall. Then, a read-depth count per window, 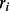, is calculated.

Third, a GC content normalization is applied to each window *i* based on the following adjustment: 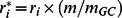, where *m* is the median of read-depth counts across all windows and *m_GC_* is the median of read-depth counts across all windows with the same GC content as the actual *i*th window [15]. To liken the data to a normal distribution, we further use the square-root transformation and use the global median as a shift parameter towards 0: 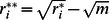. Based on the formula, values above/below 0 (or a user defined threshold, which we empirically set at 0.4/−0.4) are interpreted as copy number gains/losses.

Fourth, an adaptation of the Genome Alteration Detection Algorithm (GADA) [37] is used to detect copy number variable genomic segments. This is possible because the adjusted read-depth signal 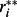 is analogous to the data properties of the log relative intensities obtained from CGH or SNP array experiments to identify CNV calls. The GADA algorithm exploits the use of piece wise constant vectors to represent CNVs and sparse Bayesian learning to detect the breakpoints. This algorithm has been shown to improve the computational speed among other algorithms, which is desirable given the huge volume of data obtained in NGS-based studies.

### 1.5 Defining and Interpreting Structural Variants

Finally, the results from the two previous modules are combined to define and interpret four different types of SVs, classified into two main categories: (a) unbalanced SVs, including those rearrangements that are accompanied by significant increase or decrease of the depth-of-coverage (involving deletions and gains of genetic material); and (b) balanced SVs, including those rearrangements with copy number neutral values in depth-of-coverage as described below.

#### (a) Unbalanced SVs

**Figure 1:**
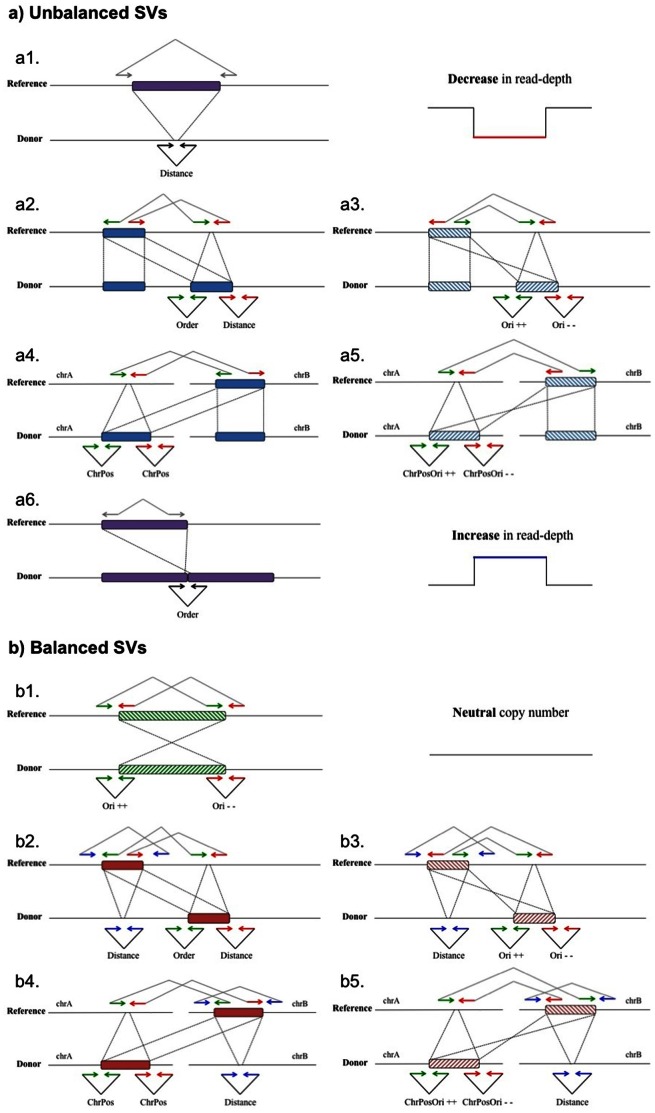
Combination of different types of anomalous read-pair alignments together with read depth (RD) pattern to define four different categories of structural variants (SVs). Case a.1 plus a decrease in RD represents a deletion. Cases a.2, a.3, a.4, a5 and a.6 together with an increase in RD represent different types of copy number gains in terms of the co-localization of the copy. Cases a.2 and a.3 represent a copy inserted within the same chromosome, were in case a.3 the copy is inserted in an inverted orientation. Analogous cases a.4 and a.5 represent straight or inverted insertions of copies in another chromosome. Case a.6 represents tandem duplications. Case b1 corresponds to an inversion. Cases b2 and b3 correspond to an intra-chromosomal translocation, but in case b3 the translocated region is inserted in inverted orientation. Similarly cases b4 and b5 correspond to inter-chromosomal translocations.

A deletion is defined by the overlap of genomic regions within breakpoint predictions from distance-pairs with copy number losses from the read depth analysis (Figure 1-a.1).

The definition of a copy number gain depends on the nature of the rearrangement. In the case of tandem duplications, genomic regions within breakpoint predictions from *order*-pairs must overlap with copy number gains in read depth analysis (Figure 1-a.6). A copy number gain of a region within the same chromosome but not in tandem is found combining *distance*- and *order*-pairs clusters with an increase of coverage depth of the copied region (as shown in Figure 1-a.2). If the copied region is inserted in inverted orientation, it is identified by a combination of *ori-*pairs clusters, one comprising *ori*-pairs anchored to the forward strand and the other on the reverse (Figure 1-a.3), and an increase in read depth analysis of the copied region. When the copy is inserted into a different chromosome, the combinatorial process is similar as those just explained, but the clusters combined in these cases are based on *chrpos*- or *chrposori*-pairs (Figure 1-a.4 and 1-a.6). In all cases, *PeSVFisher* defines the copied region and the insertion breakpoint.

#### (b) Balanced SVs

Inversions are defined by their two breakpoints: breakpoint predictions from clusters of ori-pairs anchored to the forward strand should be equal to breakpoint predictions from clusters of ori-pairs anchored on the reverse strand (Figure 1-b.1).

The definition of neutral intra- and inter-chromosomal translocations is based on the combination of three cluster types, also accompanied by a copy number neutral pattern from the read depth analysis. If a genomic region has been translocated to another location within the same chromosome, two *distance-* and one *order-*cluster should be combined (Figures 1-b.2). When the translocated region is inserted in an inverted orientation, the combination should include *distance-* and *ori*-clusters as shown in Figure 1-b.3. If the region is translocated into another chromosome, the combinatorial process comprises *distance-* and *chrpos-* or *chrposori-*clusters, depending on whether the translocation is inserted directly or in an inverted orientation (Figures 1-b.4 and 1-b.5).

### 1.6 Filtering Non-defined Structural Variant Calls (FinalCountDown)

The identification of genomic rearrangements based on PR strategies is prone to wrong predictions, especially when reads fall onto repetitive regions of the genome (i.e., simple sequence repeats, segmental duplications, and transposable elements). This is particularly important in the case of balanced SVs, as they are only supported by the PR strategy. Thus, the proportion of false positive predictions could be drastically reduced if those predictions falling within repetitive regions were filtered out. For this purpose, *PeSV-Fisher* includes an optional module called FinalCountDown, which searches for and removes those rearrangements where at least one of the breakpoints falls within repetitive elements of the genome, if these rearrangements are not supported by RD or by a combination of different types of PR aberrant clusters as described in the previous step. The repetitive elements considered in this step are: (1) segmental duplications (SDs), (2) simple sequence repeats (SSRs), or (3) low-divergent transposable elements (TEs) [38] (defined as those transposable element insertion sites that share >90% identity at the nucleotide level with the corresponding family consensus sequence). SSRs, SDs, and TEs GRCh37 coordinates are obtained from the UCSC Genome Bioinformatic Browser (http://genome.ucsc.edu/).

However, since there is evidence that large blocks of sequence homology (or SDs) or shorter common repeat sequences overlap with a relatively high percentage of SV breakpoints [12], the removed rearrangements could still indicate valid structural variants. Therefore, the FinalCountDown module provides one final file with putative rearrangements that passed all filters, and three files with putative rearrangements that did not pass a certain filter (those in SDs; those not in SDs but in SSR; those not in the previous two but with low-divergence from a TE). Finally, the TE-identity of the rearrangements that passed all filters is also provided.

Figure S1 shows a schematic representation of the complete process for the SV detection, interpretation and classification, and the final organization provided by *PeSV-Fisher* for the results is summarized in Figure S2.

## Results and Discussion

### Targeted Re-sequencing for Evaluating the Performance of *PeSV-Fisher* on Two Individuals with Chronic Lymphocytic Leukaemia

The research presented in this study has been approved by the “Comité Ético de Investigación Clínica” (CEIC, Hospital Clínic de Barcelona). All patients gave informed consent for their participation in the study following the International Cancer Genome Consortium (ICGC) guidelines [39]. All participants provided a written informed consent to take part in this study, which was also approved by CEIC (Hospital Clínic de Barcelona) to enter in the ICGC-Chronic Lymphocytic Leukemia Genome Project (http://www.cllgenome.es/). All patients submitted to mutational screening and clinical validation gave their informed consent in agreement with an Institutional Review Board-approved informed consent for genetic studies. All clinical investigation was conducted according to the declaration of Helsinki.

We first studied the behaviour of *PeSV-Fisher* at fine-scale breakpoint resolution in all the different types of aberrant clusters the tool is able to detect. Our aim was to evaluate the different types of breakpoint predictions by PR strategy rather than the final variant definitions. Given the nature of the genome and of NGS data (such as high percentage of repetitive regions and short read lengths), mapping programs generate a large amount of ambiguous alignments, leading to a similarly large amount of false positive breakpoint calls and thus false positive SV predictions by the PR strategy. Although *PeSV-Fisher* integrates PR and RD strategies to define a SV, balanced SVs (inversions and translocations) can only be detected by combining breakpoints from different aberrant clusters predicted by PR. Therefore, reliable aberrant cluster breakpoints are key to further define a reliable SV, especially in the case of balanced SVs.

The performance of *PeSV-Fisher* was evaluated in terms of true positive (TP) and false negative (FN) rates. To this end, we studied both normal and tumour DNA from one previously published patient with chronic lymphocytic leukaemia, case CLL2, from the Chronic Lymphocytic Leukaemia Genome Project (CLL-GP) [40]. Detailed information about sample collection and processing, biological characteristics of the tumour sample and the whole-genome sequencing (WGS) protocol for short-insert library construction are provided in a previous study [40]. Sequencing coverage statistics are shown in Table 1.

**Table 1:** Whole-genome sequencing data statistics.

		Readlength	Insert size			#Reads	Seq.coverage	#Reads	Seq.coverage
			1^st^ percentile	Median	99^th^ percentile	Unpaired+paired-reads		Paired-reads	
**CLL2**	**Tumour cells**	∼95	112	271	400	1.178.606.714	38.7x	1.138.687.234	37.4x
	**Normal cells**	∼95	78	261	370	1.161.111.680	38.1x	1.120.131.088	36.8x
**CLL16**	**Tumour cells**	∼95	84	264	405	1.377.406.890	45.2x	1.355.526.562	44.5x
	**Normal cells**	∼95	68	247	402	1.364.685.267	44.8x	1.322.094.298	43.4x
**YRI**	**NA19240**	∼40	42	186	966	2.328.156.503	32.2x	2.178.558.262	30.3x

To evaluate the TP rate of our method we collected aberrant cluster calls from the tumour sample and constructed different scenarios based on the set of confidence parameters that influence the reliability of breakpoint predictions. These parameters are: phred-scaled quality scores; *Q*, which evaluate the mappability of the read and the base call accuracy; the number of read-pairs supporting the aberrant cluster; and the length of the detected variant, which is taken into account in all cluster types except for *chrpos*- and *chrposori-*clusters. In particular, we considered two levels for *Q*: clusters with at least one RP where both reads have 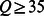 (more than 99.9% of base call accuracy), and clusters with 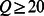 (99% of base call accuracy) excluding those potentially involved in the first *Q* level. For the number of RPs supporting each cluster, we considered 4 categories: high [10–50), medium [5–10), low [1–5) and special, in the sense that the number of RPs is significantly larger than the average sequence coverage, 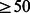. Finally, the different variant lengths categorizations were: high [1 kb –1 Mb), medium [500 bp –1 kb) and special, 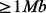. Consequently, we designed 24 scenarios that reflect the spectrum of RP based aberrant clusters obtained by *PeSV-Fisher*.

Breakpoints of the clusters integrated in these scenarios that do not contain any type of the repetitive sequences after the FinalCountDown analysis, were re-analysed by a targeted re-sequencing approach (Supporting Information S1). Note that the criterion to re-sequence only those clusters with “clean” breakpoints is due to the incapability to design specific targets for repetitive sequences. We then applied the PR strategy of *PeSV-Fisher* and an independent split-read analysis using the GEM algorithm [41] on the re-sequencing data in order to establish the TP rates of our tool conditioned on the different reliability parameters.

**Figure 2:**
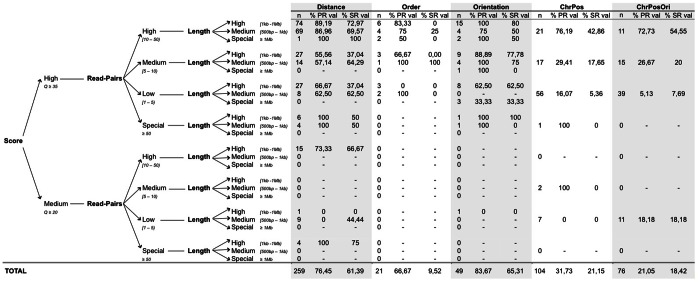
Performance of *PeSV-Fisher* based on the analysis of a high-coverage sequenced blood-cancer genome. Results according to different scenarios based on the set of confidence parameters that influence the reliability of breakpoint predictions of clusters made by anomalous alignment read-pairs. These are phred-scaled quality scores *Q*; the number of read-pairs supporting the aberrant cluster; and the length of the potential variant call. For each scenario and cluster type are represented the number of breakpoints captured by target re-sequecing of *PeSV-Fisher* calls from whole genome sequencing (n), percentage of breakpoints validated by target-re-sequencing using the paired-reads strategy (%PR) and the percentage of breakpoints validated by target-re-sequencing using split-reads analysis (%SR).

We captured the two breakpoints of 509 potential SVs, corresponding to 259 *distance*-clusters, 21 *order*-clusters, 49 *ori*-clusters, 104 *chrpos*-clusters, and 76 *chrposori-*clusters. Using the PR strategy we confirmed the two breakpoints of 76% of *distance*-clusters, 67% of *order*-clusters, 84% of *ori*-clusters, 32% of *chrpos*-clusters, and 21% of *chrposori*-clusters, while by split-reads we validated 61%, 10%, 65%, 21%, and 18%, respectively (Figure 2). The efficiency of breakpoint capture was very low in the scenarios where clusters had poor *Q* values, which could be explained by the presence of some types of repetitive sequences in the breakpoints not included in the FinalCountDown module, or by sequencing/mapping errors. Hence this only allows us to be confident with clusters containing at least one RP with 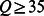, and to realize the importance of this value for the reliability of a SV even in those rearrangements that do not contain repetitive sequences in the breakpoints.

As expected, the higher the number of RPs pointing to the same alignment aberration, the more confident the results are. This is especially important in *order*-, *chrpos-*, and *chrposori-*clusters (Figure 2). In the category of special number of RPs, which includes those clusters with significantly more RPs than expected by average coverage rate, the re-sequencing approach failed to capture them, mainly because these breakpoints might also fall into specific types of highly repetitive or highly polymorphic regions not included in the FinalCownDown filtering module. Finally, according to our validation results the predicted length of the variant indicated by the anomalous RPs is also important for the reliability.

To evaluate the ability of the *PeSV-Fisher* to detect real complex SVs, we further searched for some examples of SVs that had been defined in sample CLL2 whose breakpoints have been completely validated by our targeted re-sequencing approach. Two interesting examples are further detailed in Table S1: an inter-chromosomal copy-paste event involving two different clusters of *chrposori* supported by a significant increase of RD and an intra-chromosomal cut-paste event formed by two *ori* clusters that overlapped with a *distance* cluster without a significant increase or decrease of RD.

In order to establish FN rates, we extracted breakpoints that were detected only in the tumour sample, i.e. *a priori* somatic breakpoints, and we asked if the breakpoints were also found in the normal sample using the targeted re-sequencing approach. We obtained a set of targets that should be free of variant breakpoints in the normal genome based on the WGS analysis; therefore if we detect the same breakpoint in the normal sample as in the tumour, this is defined as a FN.

Previous analyses of sample CLL2 by WGS showed that the tumour sample contains, excluding antigen receptor loci, only 6 somatic SVs [40], which were all called by *PeSV-Fisher* and validated as somatic by targeted re-sequencing. Thus, in order to establish more significant FN rates, we applied the PR strategy of our tool to another CLL case from the CLL-GP (CLL16, unpublished data). In this case, we detected by whole-genome sequencing and validated by target re-sequencing 42 clusters of potential SVs in the tumour sample, and only 5 (FN = 11.9%) of the putative somatic variants were detected in the normal tissue using the target re-sequencing approach. Information about sample collection and processing, biological characteristics of the tumour sample and the whole-genome sequencing (WGS) and targeted re-sequencing protocols are provided in Supporting Information S1.

### Comparative Analyses in a 1000 Genome Project Trio Using Other Approaches

We applied *PeSV-Fisher* to the WGS data from the daughter of the Yoruba high-coverage trio included in the 1000 Genomes Project (1000 GP) dataset (NA19240). For the analysis, we considered different paired-end libraries generated by Illumina with insert sizes ranging from 100 to 600-bp, obtaining ∼30x sequencing coverage (Table 1). This same sample is analysed in the publication of Mills *et al.* [5], which reports a complete list of deletion/insertion calls made by different institutions on the high-coverage trios and low coverage sample sets (1000 GP pilot project). From this publication, we obtained the non-redundant set of deletion calls (> = 50 nt) for high coverage trios that were either validated through the use of PCR, assembly of breakpoints (ASM), sequence capture arrays (CAP array) and a high-resolution array-CGH platform (downloaded from: ftp://ftp.1000 genomes.ebi.ac.uk/vol1/ftp/pilot_data/paper_data_sets/a_map_of_human_variation/trio/sv/(trio.2010_10.deletions.sites.vcf)).

**Figure 3:**
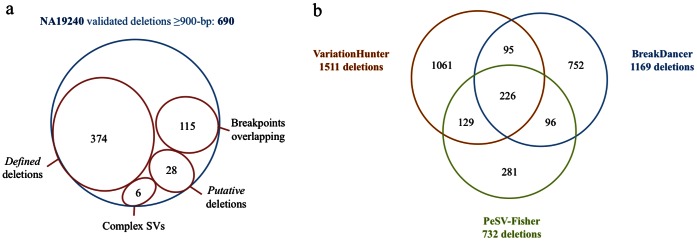
Sensitivity analysis and comparison with results from other SV prediction methods based on the analysis of the Yoruba daughter (NA19240) from the high-coverage trio from the 1000 Genomes Project dataset. (a) 90% based of the overlap of *PeSV-Fisher* calls with the non-redundant set of deletions from the publication of Mills *et al.* [5] that were validated by PCR and/or assembly. *Breakpoints* overlapping indicate *distance* clusters with predicted overlapping breakpoint ranges from each end of the variant; (b) three-way comparison of the deletion calls made by *PeSV-Fisher*, BreakDancer and VariationHunter. The analysis is carried out using a 99% of reciprocal overlap.

To examine the sensitivity of *PeSV-Fisher* on true deletion calls with well-defined breakpoints, we selected the PCR and/or ASM validated deletions ≥900-bp from the validated deletions list, independently of which algorithm was used for calling, and we checked the overlap with our calls. This 900 bp cut-off was selected based on the variability of the insert sizes in the combined libraries used. Figure 3a shows the overlap of our calls with these validated variants. *PeSV-Fisher* detected 523 distance-clusters overlapping with the validated list, which is similar to those detected by BD (541) or VH (558). Nonetheless, only 374 of these 523 distance-clusters were finally classified as *deletions*, showing a significant decrease of RD and non-overlapping any other PR clusters. Of the remaining 149, in 6 cases the variant definition classified them as other than deletions (two copy-paste and two cut-paste events) and in 28 cases as putative deletions. Unsurprisingly, of these 6 variants not classified as deletions by PeSV-Fisher, 5 contain breakpoints located in highly repetitive regions close to telomeres. Sites with high sequence diversity are prone to generate multiple and similar breakpoint predictions by several types of anomalous RPs, reflecting the complexity of the sequence analysis. This reveals the importance of the combination of two types of strategies like PR and RD to correctly interpret and define the individual status for the high polymorphic loci and the complex genomic events. For the last 115 *distance* calls, the predicted breakpoint ranges for each end of the variant were overlapping and we usually discard these calls as unreliable. This overlap is probably due to the high variability in the libraries insert sizes.

We also compared our performance with that of BreakDancer and VariationHunter, checking the overlap of our calls with those of these two algorithms obtained from a Supplementary Table of Mills *et al*. [5] (downloadable from http://www.nature.com/nature/journal/v470/n7332/extref/nature09708-s5.zip and corresponding to Washington University –WU- and University of Washington –UW- indicators, respectively). We did not compare to algorithms that do not use the PR strategy, as they are conditioned to the analysis of unbalanced SVs, or to data generated by unpublished algorithms. Figure 3b summarizes the overlap of the predicted *defined* deletions made on the Yoruban daughter NA19240 by the three different algorithms. We considered that two different algorithms were calling the same deletion if there was a 99% reciprocal overlap. With this stringent value we ensured that the overlap measure was symmetric. Each algorithm uniquely called a large number of calls; however a fraction of these calls might be due to false positives or different breakpoint accuracies. Another explanation might be the different size distribution patterns of deletion calls reported by the three algorithms. However, the three algorithms show the expected peak that corresponds to the 6 kb average sizes of full-length LINE-1 human retrotransposons [42] (data not shown).

### Computational Considerations

As argued by [43], NGS data imposes challenges on procedures applied for storage and analysis of data. The computer memory is usually a limitation to the analysis and, moreover, the run time scales approximately linearly with the number of reads. A plausible alternative is to search for modular code methods or to split the data in order to divide the process in multiple threads. Thus, the current tendency is to parallelize processes and use large-scale computing clusters with high numbers of cores. However, *PeSV-Fisher* can be launched on either a cluster or a workstation. This is achieved by splitting the data by chromosomes and by making classes with low coupling between them. This approach can be easily parallelized. Furthermore, we paid special attention to memory balancing using sorting strategies that directly manipulate files.

To exemplify the applicability of *PeSV-Fisher* to processing of large datasets we recorded basic timing information for processing of whole-genome paired-end data from medium to high coverage (16x to 45x). PeSV-Fisher was launched in multithreading mode (5 cores for read depth process and 5 cores for the definition of anomalous RPs plus clustering processes by sample) in a workstation with 12 cores and 48GB of memory under a Linux environment. It required approximately an average of 3915 CPU minutes for processing an average of 1.377 million reads generated by an Illumina platform using a median fragment size of 264. Two samples with medium coverage (16x–20x) were completed in parallel mode in approximately 265 minutes and two samples with high coverage (45x) needed 805 minutes. The memory by thread in the medium coverage sample was about 0.1–0.4 GB during the longest part of the execution, but displayed peaks of 1.1–1.4 GB at the sorting step in definition of anomalous RPs plus clustering modules. The memory used for the samples with high coverage was similar but peaks were in the range of 1.7–2.4 GB.

The analysis time for the clustering processes present a high dependency on hard drive speed; however, the analysis time for read depth processing is more dependent on the CPU speed. So, the read depth and definition of anomalous RPs plus clustering modules for each sample can be launched simultaneously for a better performance and resource utilization.

Further computational aspects are given in the Supporting Information S1.

### Conclusions

Despite constantly ongoing development of algorithms for the detection of SVs, the complexity of NGS data and of the human genome hampers SV discovery as well as the understanding of the subsequent biological interpretation and consequences.

We presented here our computational tool, *PeSV-Fisher*, for the detection and characterization of SVs using NGS data. We employed a new strategy to correctly interpret SV calls generated by paired-reads approaches, combining different types of aberrantly aligned read-pairs, and a read depth strategy. By doing so, *PeSV-Fisher* is capable of defining five general types of structural rearrangements (deletions, gains, intra- and inter-chromosomal translocations, and inversions), but goes one step further in their definition with the interpretation of co-localization of identified aberrant calls. Additionally, *PeSV-Fisher* keeps information concerning the orientation of aberrantly aligned read-pairs. For example, the positional effect of a gene copy in any region of the genome could be different if this copy is inserted in one orientation or another.

*PeSV-Fisher* does not take into account the diploid nature of the human genome, although it is an aspect recently being considered in strategies to solve the co-localization of more than two variants at the same locus [26]. However, our tool has been designed especially for the analysis of cancer genomes, where clonal mosaicism, which is defined as the coexistence of cells with two or more distinct genotypes within an individual, has been observed [44], [45]. Interestingly, clonal mosaicism has also been described in aging genomes [44], which means that it not only happens in cancer genomes, but also is a common phenomenon appearing throughout the genome evolution within an individual.

Finally, we should highlight the good performance of the tool in terms of sensitivity and true positive and negative rates, with a reasonable computational cost in a single workstation.

## Supporting Information

Figure S1 **Workflow of structural variants definition module of** ***PesV-Fisher*** **.**
(TIFF)

Figure S2 **Output files organization.** ***PeSV-Fisher*** **generates a general** ***Results*** **folder containing three sub-folders called** ***clusters, dofc*** **and** ***sv,*** **which contain results from PR strategy, RD strategy and the results from the combination of both strategies, respectively.**
(TIFF)

Table S1 **Examples of complex structural variants defined by** ***PeSV-Fisher*** **in the chronic lymphocytic leukemia sample CLL2 and validated by re-sequencing approach.**
(XLSX)

Supporting Information S1 (DOC)
