# Correction: PeSV-Fisher: Identification of Somatic and Non-Somatic Structural Variants Using Next Generation Sequencing Data

**DOI:** 10.1371/annotation/6444bc1a-3501-482e-8cbe-a27ebc66b153

**Published:** 2013-11-06

**Authors:** Geòrgia Escaramís, Cristian Tornador, Laia Bassaganyas, Raquel Rabionet, Jose M. C. Tubio, Alexander Martínez-Fundichely, Mario Cáceres, Marta Gut, Stephan Ossowski, Xavier Estivill

The following information was missing from the Funding section: the Spanish Ministry of Economy and Competitiveness through the Instituto de Salud Carlos III within the ICGC CLL-Genome Project.

In 1.1 Defining Anomalous Read-pairs and in 1.2 Clustering Procedure and Breakpoint Predictions, the number 1 was mistakenly added in front of the greek letters α and σ. 

