# Supplementary figures and images for: PeSV-Fisher: Identification of Somatic and Non-Somatic Structural Variants Using Next Generation Sequencing Data
Source: PLoS One. 2013 May 21;8(5):e63377. doi: 10.1371/journal.pone.0063377 (PMC3660373; doi:10.1371/journal.pone.0063377)

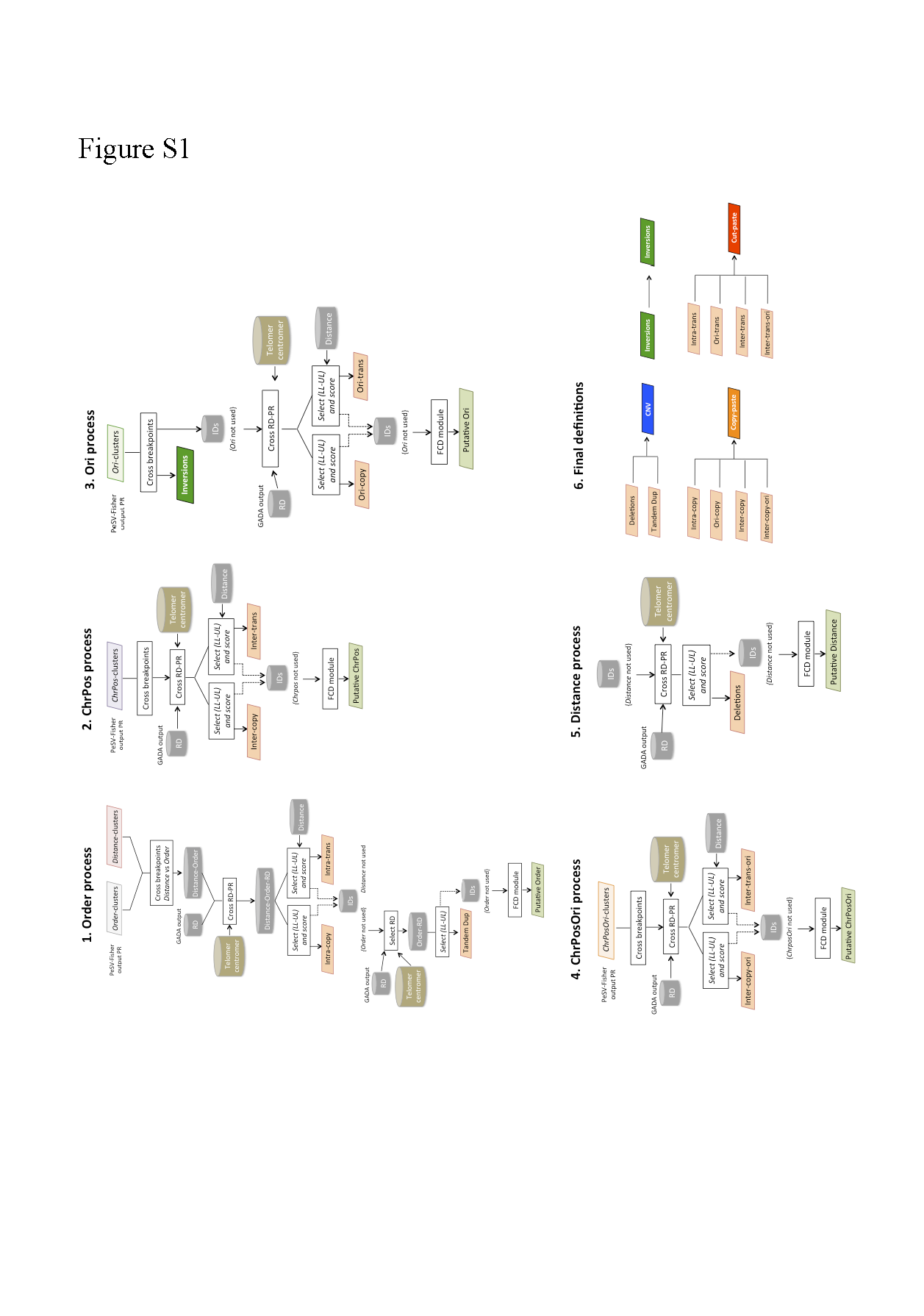

Supplement: Figure S1 — Workflow of structural variants definition module of PesV-Fisher . (TIFF) [file pone.0063377.s001.tiff]

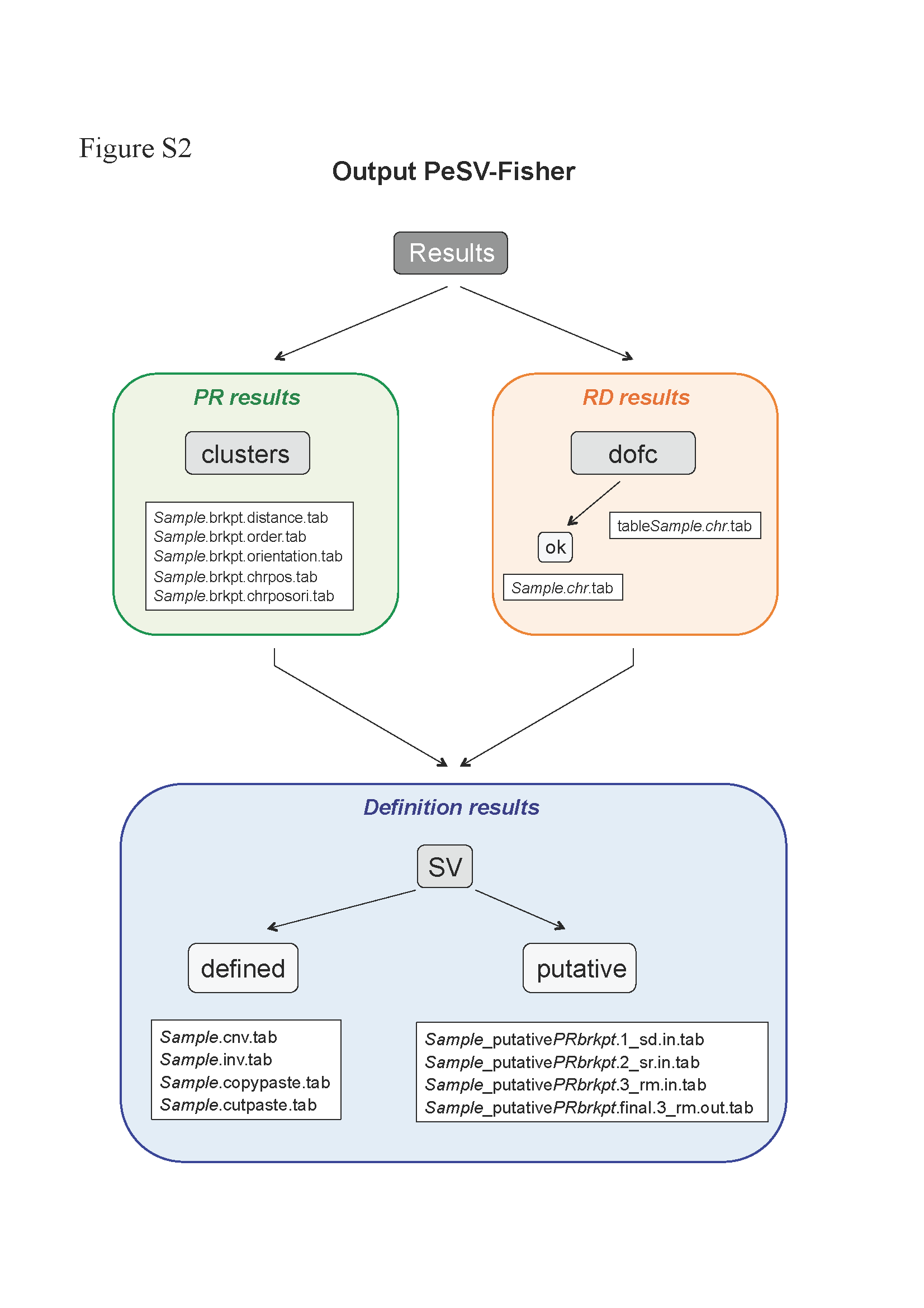

Supplement: Figure S2 — Output files organization. PeSV-Fisher generates a general Results folder containing three sub-folders called clusters, dofc and sv, which contain results from PR strategy, RD strategy and the results from the combination of both strategies, respectively. (TIFF) [file pone.0063377.s002.tiff]
