# Supplementary material for: PeSV-Fisher: Identification of Somatic and Non-Somatic Structural Variants Using Next Generation Sequencing Data
Source: PLoS One. 2013 May 21;8(5):e63377. doi: 10.1371/journal.pone.0063377 (PMC3660373; doi:10.1371/journal.pone.0063377)
Supplement: Supporting Information S1 — (DOC) [file pone.0063377.s004.doc]

**SUPPLEMENTARY INFORMATION S1**

**Targeted Enrichment Sequencing**

Targeted DNA enrichment was performed with SureSelect custom capture system (Agilent Technologies, Santa Clara, CA) according to the SureSelect Target Enrichment protocol and sequenced on an Illumina HiSeq2000 instrument, following manufacturers' protocols. Briefly, 3 μg of human genomic DNA were sheared using a Covaris E220 to a size of roughly 150bp. The purified samples were end repaired, adaptor ligated and 6 cycles of PCR were applied. In the following step the PCR product was hybridized to the Agilent bait capture kit for 24h for the enrichment and subsequently amplified by 12 cycles of PCR. Resulting libraries were sequenced on Illumina HiSeq2000 flowcells in pools of 3 samples per sequencing lane, generating 50-80 million paired end sequence reads of 76 base pairs per sample. Image analysis, base calling, and base call quality was generated during the run with the Illumina HiSeq Real Time Analysis (RTA 1.13.48) software with default parameters. FASTQ files containing sequence information and quality scores for each base call were exported for further analysis.

Sequence reads were mapped to the human reference genome (GRCh37/hg19) using Burrows-Wheeler aligner (BWA) and the resulting unmapped reads were extracted for further split-read analysis using GEM algorithm.

**Computational considerations**

**1.1 General workflow**

Samtools

Bam

BD

**RD:** applies read depth strategy**, PR:** includes definition of anomalous read-pairs, clustering procedure and breakpoint prediction, **SV:** defines of structural variants**, FCD:** filters structural variant calls.

**2.2 Multithreading description of execution**

Example of the multithreading option to run the process with tumour and normal paired samples: the main process is split in two threads, and each thread is divided by read-depth module (RD) and definition of anomalous read-pairs plus clustering processes too (PR). RD and PR parts are launched at the same time by sample for a better performance and resource utilization. The outputs from RD and PR parts are recovered from database for the SV definitions.

PR

RD

normal sample

tumor sample

SV + FCD

**2.3 Multithreading time of execution**

The times are acquired using a Workstation 48GB, x12 cores with Fedora OS. The time depends on hard drive speed and network if the system is a distributed environment. The SV+FCD depend on the efficiency of your mysql database.

| **Cores** | **16x tumour + 20x normal samples** | | | | **45x tumour + 45x normal samples** | | | |
| --- | --- | --- | --- | --- | --- | --- | --- | --- |
| **(5+5)x2** | Chr1 | Mean | Total | Mem | Chr1 | Mean | Total | Mem |
| **Samtools** | 60 | 32 | 160 | ~NaN | 170 | 96 | 480 | ~NaN |
| **Clustering** | 95 | 51 | 255 | 0.2 | 280 | 155 | 780 | 0.4 |
| **RD** | 55 | 28 | 190 | 0.2 | 106 | 45 | 360 | 0.2 |
| **SV+FCD** | 10 | 10 | 10 | ~NaN | 15 | 15 | 15 | ~NaN |
| **Total** | 105 | 61 | 265 | 0.2 | 295 | 170 | 805 | 0.4 |

The first column represents the time for the longest chromosome per sample with medium coverage (16x-20x), the second is the mean time per chromosome, the third is the total time for both samples, normal and tumour, and the forth represents the mean memory used per sample with medium coverage samples (analogous for the subsequent columns based on 45x samples).

**References**

1. Li H, Durbin R (2009) Fast and accurate short read alignment with Burrows-Wheeler transform. Bioinformatics 25: 1754-1760.

2. Marco-Sola S, Sammeth M, Guigo R, Ribeca P (2012) The GEM mapper: fast, accurate and versatile alignment by filtration. Nat Methods.

**Supplementary Figures**

**Figure S1. Workflow of the SV definition module**

**Figure S2. Output files organization**. *PeSV-Fisher* generates a general *Results* folder containing three sub-folders called *clusters, dofc* and *sv,* which contain results from PR strategy, RD strategy and the results from the combination of both strategies, respectively.
